# Effectiveness of Pre-operative Respiratory Muscle Training versus Conventional Treatment for Improving Post operative Pulmonary Health after Coronary Artery Bypass Grafting

**DOI:** 10.12669/pjms.36.6.2899

**Published:** 2020

**Authors:** Wajeeha Sahar, Noor Ajaz, Zulfiqar Haider, Anjum Jalal

**Affiliations:** 1 Wajeeha Sahar, Department of Physiotherapy, Faisalabad Institute of Cardiology, Faisalabad, Pakistan; 2 Noor Ajaz, KKT-Orthopedic and Spine Center, Faisalabad, Pakistan; 3 Zulfiqar Haider, FRCS-CTh. Punjab Institute of Cardiology, Lahore, Pakistan. Department of Cardiac Surgery, Punjab Institute of Cardiology, Jail Road, Lahore, Pakistan; 4 Anjum Jalal, FRCS-CTh. Office of the Executive Director, Faisalabad Institute of Cardiology, Faisalabad, Pakistan

**Keywords:** Cardiopulmonary Physiotherapy, Inspiratory Muscle Training, Breathing exercises, post-operative pulmonary health, incentive spirometry,6-minute walk test, Coronary artery bypass grafting

## Abstract

**Objective:**

To evaluate the Effectiveness of Pre-operative Respiratory Muscle Training versus Conventional Treatment for Improving post-operative pulmonary health after Coronary Artery Bypass Graft Surgery (CABG).

**Methods:**

A Prospective Randomized clinical trial was performed on sixty patients who underwent elective CABG at Faisalabad Institute of Cardiology. At the time of admission all patients were subjected to 6-minutes’ walk test (6MWT) as baseline. The subjects were then divided into two groups. The Group-I was subjected to respiratory muscle training whereas the Group-2 received the routine preoperative care. The 6-minute walk test (6MWT) was then repeated a day before surgery (pre-operative) and before discharge (post-operatively). Duration of post-operative mechanical ventilation, oxygen therapy and hospital stay were also noted as outcome measures of this study.

**Results:**

The pre-operative and post-operative readings showed that the patients in the interventional group performed better than the control group in their 6MWT with P-value of less than 0.05. Similarly the interventional group had shorter duration of mechanical ventilation, dependence on oxygen therapy and postoperative hospital stay as compared with the control group showing P-values below 0.05.

**Conclusion:**

The results showed that respiratory muscle training results in improved postoperative functional capacity and reduces of hospital stay.

## INTRODUCTION

The available literature supports that one of the major cause of post-operative pulmonary problems which increases the duration of mechanical ventilation and oxygen therapy was poor preoperative pulmonary health. Therefore, there is need to find if any improvement of pre-operative pulmonary health in patients selected for Coronary Artery Bypass Grafting has impact on outcome of surgery. The purpose of this study was to evaluate the effectiveness of preoperative inspiratory muscle training in patients undergoing Coronary Artery Bypass Grafting and assess their pulmonary status after surgery.

## METHODS

Both male or female patients within age group of 40-65 years planned for coronary artery bypass grafting and who were willing to participate in the study were included. The patients showing inconsistency with initial stages of exercise rehabilitation were excluded. Similarly patients suffering from asthma, COPD, interstitial lung disease, endocrine abnormalities such as hyper or hypothyroidism, neurological disability affecting respiratory rate, left ventricular ejection fraction <40%, concomitant valve disease, dysrhythmias, pacemaker dependency, anemia and obesity were also excluded from the study.

A total of 60 patients were included in study and they were randomized to Group-I as Intervention Group and Group-2 as Control group. The mean age in group Group-I was 53.97 years with standard deviation of 7.29 years while the mean age in Group-2 was 54.13 years with standard deviation of 4.17 years. The difference between the mean age of both groups was not significant (P=0.91). The gender distribution in both groups was slightly disproportionate. The Group-I had 27 male and three female patients whereas the Group-2 had 22 male and eight female patients. However, the difference in gender distribution of both groups was not significant as the p-value calculated by chi-square test was 0.09.

### The Null Hypothesis was defined as

“There is no significant difference between respiratory muscle training and conventional treatment”.

### While the alternate Hypothesis was defined as

“There was significant difference between respiratory muscle training and conventional treatment”.

Modified Healthy Heart Questionnaire (HHQ-GP-1) was used as a screening tool and The 6MWT was performed as a baseline in Group-I and Group-2 on the day of admission. The distance covered (feet), heart rate and oxygen saturation were noted after the 6-minutes walk. Portable finger Pulse Oximeter was used for assessment of oxygen saturation and heart rate. After this the patients in the Group-I received respiratory muscle training (RMT) which consisted of the incentive spirometer (IS), diaphragmatic breathing, segmental breathing movement (lateral costal expansion, posterior costal expansion, apical costal expansion exercises) and huff-coughing techniques. A total of 28 sessions of respiratory muscle training were held in Group-I for >15 minutes at a constant low speed for each patient. The patients in Group-2, received conventional treatment which includes deep breathing. The above mentioned 6-minute walk test was then repeated a day before the surgery (preoperative values) and then at the time of discharge after surgery (postoperative values).

Statistical analysis was performed by using SPSS version 20. Independent Sample T test was applied on numeric data. The difference in the gender distribution was evaluated by Chi-square test. The P-value of 0.05 or below was considered as significant.

## RESULTS

The results showed that there was significant difference between interventional and control group in their distance covered in six minute walk test, oxygen saturations and heart rate. Similarly duration of mechanical ventilation, dependence on oxygen therapy and the length of post-operative hospital stay were also statistically different in both groups.

The Table-I summarizes the results of both groups. It shows that there was no significant difference among both groups in the distance covered in 6-minutes’ walk test at baseline (P = 0.481). However, there was significant difference in their pre-operative (P = 0.005) and post-operative readings (P = 0.002). This means that the patients who received preoperative muscle training did better in 6-minutes’ walk test. Similarly, in the heart rates measured after 6-minute walk test there was insignificant difference among both groups at baseline (P = 0.241) but the difference in preoperative (P = 0.046) and post-operative (P = 0.005) readings became significant. The slower heart rates in muscle training group noted, is an indicator of better functional capacity. In case of oxygen saturation, there was insignificant difference among both groups at baseline (P = 0.205) but there was significant difference preoperatively (P = 0.002) and before discharge (P = 0.001). Here again the patients in the intervention group had better oxygen saturations. Similarly it is clear that there was significant difference between Interventional and Control Group in the duration of mechanical ventilation (P = 0.001) and in the duration of postoperative oxygen therapy (P = 0.001). Both of these durations were shorter in the intervention group. This naturally resulted in shorter postoperative hospital stay, which was statistically significant (P = 0.001).

**Table-I:** Results.

Observations/Test	Timing	Interventional Group		Conventional Group		P-Value

		Mean	Standard Deviation	Mean	Standard Deviation	P-Value
Distance Covered in 6 MWT (feet)	Baseline	441.47	217.554	404.80	181.380	0.481
	Pre-op	513.80	183.171	376.10	181.705	0.005
	Before Discharge	369.73	140.186	250.80	148.260	0.002
Heart Rate after 6MWT (beats/min)	Baseline	77.67	12.861	81.27	10.544	0.241
	Pre-op	76.50	5.400	81.13	11.246	0.046
	Before Discharge	79.90	6.397	86.47	10.679	0.005
Oxygen Saturation after 6MWT (%)	Baseline	97.40	1.868	96.73	2.149	0.205
	Pre-op	96.87	2.389	95.13	1.737	0.002
	Before Discharge	95.70	4.195	92.70	2.277	0.001
Ventilation Duration (hours)	Post-op	5.13	1.008	6.17	1.315	0.001
Oxygen Therapy (hours)	Post-op	54.20	7.993	62.13	10.139	0.001
Post op Hospital Stay (days)	Post-op	5.40	0.814	6.27	1.112	0.001

## DISCUSSION

The arterial oxygen saturation levels are known to improve after inspiratory muscle exercise pre-operatively and before discharge.^1^ Preoperative inspiratory muscle training is found to result in improved inspiratory muscle power and better alveolar-arterial oxygen gradients.^2^ The results of study by Bavarsad et al showed that, although inspiratory muscle training did not increase the SPO2 level after exercise in patients with chronic obstructive pulmonary disease yet it prevented a decrease in SPO2 level after exercise.^3^ Therefore, it is justified to anticipate that it can lead to an increase in exercise tolerance in these patients. The use of inspiratory muscle training in the post-operative period is also known to have similar benefits. This is especially useful in chronically debilitated patient of valve diseases who have poor general health and loss of muscle mass. This benefit was demonstrated recently by Cargnin et al who performed four weeks of inspiratory muscle training after the vale surgery and studied the outcome results.^4^ They found significant improvement in 6-minutes walk test, maximum inspiratory pressure and quality of life in the group that received inspiratory muscle training.

Elkins et al have reported that inspiratory muscle training significantly improved rapid shallow breathing.^5^ This resulted in early weaning from the ventilator. Hence it is likely to reduce length of stay in ICU, dependence on non-invasive ventilatory support and total stay in hospital. The length of stay on ventilator was found to be independent of muscle training in study by Condessa et al.^6^ However, this study included an entirely different subset of non-cardiac ICU patients who were on ventilator for over 48 hours for any reason. The inspiratory muscle training was done while the patients were on ventilator and were ready for weaning. This is at variance with a conclusion of the review article by Bisset et al wherein they advised early and proactive rehabilitation of the respiratory muscles whenever possible in ICU patients.^7^ They also recommended to implement targeted and individualized training of respiratory muscles which is expected to improve quality of life of the patients. In fact, critically ill patients represent a complex group of patients and consequently the benefits of physiotherapy in them are not uniform. The Task Force of European Society of Intensive Care Medicine on Physiotherapy for Critically Ill Patients have noted discrepancies and irregularities in the reported literature and have highlighted to formulate guidelines for physiotherapy assessments and standardization of pathways for clinical decision making.^8^

There are several reports regarding the efficacy of perioperative breathing and coughing exercises in prevention of post of pulmonary complications (PPCs). Stiller et al, (1994) investigated the efficacy of 3 to 5 deep-breathing and coughing exercise in the prevention of PPCs after CABG and reported that prophylactic chest physical therapy did not prevent PPCs after CABG.^9^ Diaphragm dysfunction is a known complication of cardiac surgery. It may persist and require surgical plication of diaphragm. Kodric et al studied the role of inspiratory muscle training in such patients. They found that Inspiratory muscle training produced a significant improvement of diaphragm mobility after 12 months (P < .001). Out of 36 patient the intervention group, 41.67% experienced a partial improvement while 36.11% achieved a complete improvement and no recovery was found in 22.22%. In the control group there was no improvement in 87.5% and partial recovery in 12.5%.^10^ Early mobilization and breathing exercises are frequently employed to prevent postoperative pulmonary complications after cardiac surgery. The optimal method and frequency of deep breathing is however not known. Urell et al evaluated the effect of 30 versus 10 deep breaths hourly with positive expiratory pressure on first postoperative day of cardiac surgery. They found a significantly increased oxygenation in patients performing 30 deep breaths per hour.^11^

The finding of the present study was in accordance with Matheus et al, who showed a substantial reduction in length of stay in the coronary care unit in the study group and present study also indicated reduction in days of ICU stay.^12^ This is also supported by a similar trial by Savji et al which showed significant reduction in hospital stay and much lower anxiety levels in the control group.^13^

## CONCLUSION

It is concluded that inspiratory muscle training is effective in improving the respiratory muscle strength and functional capacity of patients elected for cardiac surgery. This training results in better pulmonary outcomes after CABG.

### Contribution of authors:

**WS:** Project concept; Data collection, Statistical Analysis and Manuscript writing

**NA:** Manuscript writing

**ZH:** Study Design

**AJ:** Supervision; Manuscript Editing

**WS** takes the overall responsibility and is accountable for ensuring that questions related to the accuracy or integrity of any part of the work are appropriately investigated and resolved.
